# Nursing Roles in Early Integration of Palliative and Supportive Care for Adults with Advanced Cancer: A Scoping Review

**DOI:** 10.3390/curroncol33060312

**Published:** 2026-05-27

**Authors:** Omar Alqaisi, Suhair Al-Ghabeesh, Hanin Masalha, Aoife Jones Thachuthara, Kurian Joseph, Patricia Tai, Edward Yu, Rashmi Koul

**Affiliations:** 1 Nursing Department, Al-Zaytoonah University, Airport Street, Amman 11733, Jordan; s.alghabeesh@zuj.edu.jo; 2 Faculty of Nursing, Hamad Medical Corporation, Doha P.O. Box 3488, Qatar; haninmasalha@yahoo.com; 3 Department of Medical Oncology, Cork University Hospital, T12 DC4A Cork, Ireland; aoife.jones3@hse.ie; 4 Devision of Radiation Oncology, Cross Cancer Institute, 11560 University Avenue, Edmonton, AB T6G 1Z2, Canada; kurian.joseph@cancercarealberta.ca; 5 Department of Oncology, University of Saskatchewan, Saskatoon, SK S7N 5A2, Canada; pat961@usask.ca; 6 Department of Oncology, Western University, London, ON N6K 3K7, Canada; eyu@uwo.ca; 7 Department of Oncology, University of Manitoba, 675 McDermot Ave., Winnipeg, MB R3E 0V9, Canada; rkoul@cancercare.mb.ca

**Keywords:** early palliative care, oncology nursing, advance directive, supportive care, nurse-led interventions, scoping review

## Abstract

As the global cancer burden rises, adults with advanced cancer face significant physical and psychosocial symptoms requiring early integration of palliative and supportive care. Nurses in oncology, emergency, and community settings are central to symptom assessment, care coordination, communication, and advance care planning, yet their roles in early integration remain underexplored. This scoping review mapped nursing contributions to early palliative and supportive care for adults with advanced cancer and described related patient, caregiver, and system outcomes. A search of PubMed, CINAHL, Scopus, and ScienceDirect was conducted for English-language studies published between January 2016 and November 2025 involving nursing-relevant interventions in early palliative or supportive care. Fourteen studies were included: trials, observational studies, qualitative research, reviews, and a meta-analysis. Six domains emerged. Early integration consistently improved quality of life and reduced symptom burden. Nurse-led interventions increased end-of-life discussions and advance directive completion. Telehealth and telephone follow-up proved feasible for symptom management. Studies noted moderate palliative competence but gaps in communication and structural support. Caregiver-focused interventions enhanced caregiver quality of life and self-efficacy. Conclusions: Nurses are pivotal in early palliative care. Expanding structured nurse-led models, strengthening communication training, and addressing organizational barriers are essential to deliver timely, person-centered care.

## Simple Summary

Cancer is a leading cause of death worldwide, and patients with advanced cancer often experience severe physical and emotional symptoms that lead to frequent emergency visits and hospitalizations. Palliative and supportive care, when introduced early rather than at the end of life, can significantly improve patients’ quality of life and reduce symptom burden. Nurses play a central role in delivering this early care through symptom assessment, care planning, telephone follow-up, and caregiver support; however, their contributions have not been comprehensively mapped. This scoping review examined 14 studies and identified six key domains of nursing involvement in early palliative and supportive care for adults with advanced cancer. The findings revealed that nurse-led interventions improve patient and caregiver outcomes, yet gaps in communication training, role clarity, and organizational support persist, which can hinder the effectiveness of these interventions and the overall quality of care provided. These results can inform future policies and educational programs aimed at strengthening nurses’ roles in delivering timely, patient-centered palliative care.

## 1. Introduction

Cancer is a leading global public health challenge, with an estimated 20 million new cases and 9.7 million deaths worldwide in 2022. For adults with advanced cancer, the integration of early palliative and supportive care alongside active oncological treatment has been shown to improve quality of life, reduce symptom burden, and, in some cases, prolong survival. Critically, nurses, as the healthcare professionals with the most sustained patient contact across oncology, emergency, and community settings, are positioned to operationalize this early integration in ways that directly affect clinical outcomes. Yet the precise scope of nursing roles in early palliative care, and the patient, caregiver, and system-level outcomes associated with these roles, has not been comprehensively mapped [1]. Adults with advanced cancer frequently experience acute symptom exacerbations and oncological emergencies requiring urgent nursing assessment and interdisciplinary management; early palliative care integration has been shown to reduce these acute care encounters by proactively addressing symptom burden and goals of care [2,3,4,5,6,7]. In the United States, cancer-related emergency department (ED) visits account for approximately 4.2% of all adult visits, with roughly 4 million visits annually [4]. A cross-sectional study of 35.5 million ED visits among cancer patients revealed that 51.6% were potentially preventable, increasing from 1.8 million in 2012 to 3.2 million in 2019 [5]. Cancer-related ED visits result in inpatient admissions at 59.7%, significantly higher than the 16.3% rate for non-cancer visits [4]. Early recognition of oncologic emergencies (OE) is critical, as delays directly worsen patient outcomes. Alsharawneh et al. demonstrated that subtle oncologic presentations result in an average 47.5 min delay in being seen, a 33 min delay in treatment initiation, a fourfold increase in intensive care unit admissions, and a three-fold rise in mortality risk [8]. Nurses, as frontline providers, are uniquely positioned to detect subtle changes such as new-onset back pain or urinary difficulties that may herald emergencies like malignant spinal cord compression [9,10]. An early palliative care trial demonstrated that early palliative care improved quality of life, reduced aggressive end-of-life care (33% vs. 54%), and was associated with longer median survival (8.9 vs. 11.6 months in the intervention vs. control group) [10].

Patients with advanced cancer are also at risk of oncologic emergencies such as metabolic (e.g., hypercalcemia, tumor lysis syndrome), hematologic (e.g., disseminated intravascular coagulation, febrile neutropenia), structural (e.g., superior vena cava syndrome, malignant spinal cord compression), and treatment-related complications, which require timely recognition and interdisciplinary management [11].

A systematic review examining 49 studies found infection as the most common OE (22/49 studies), followed by pain (20/49), dyspnea (19/49), and gastrointestinal symptoms (17/49) [12]. Nursing care encompasses continuous monitoring that includes electrolyte monitoring; pain management; infection control; and patient/family education which are well accepted by patients [13]. Concurrently, the American Society of Clinical Oncology (ASCO) recommends early integration of palliative care, ideally within eight weeks of advanced cancer diagnosis [14], and the European Society for Medical Oncology (ESMO) similarly endorses concurrent palliative care throughout the cancer trajectory [15], with a good example [16].

Despite the high symptom burden and acute complications faced by adults with advanced cancer, existing evidence on early palliative care has predominantly focused on physician-led or interdisciplinary team-based interventions, with nursing contributions remaining underexplored as a distinct subject of inquiry. While several systematic reviews have established the clinical efficacy of early palliative care integration, none have mapped the specific scope, nature, and outcomes associated with nursing roles across diverse healthcare settings. This gap is clinically significant: nurses represent the largest healthcare workforce in oncology settings and are often the providers who most consistently interact with patients and caregivers across the illness trajectory. Yet, the evidence base needed to guide nurse education, role delineation, and policy development in this area remains fragmented. Yilmaz et al. highlighted that many studies in acute and oncologic contexts do not clearly specify cancer type, stage, or treatment characteristics, limiting the transferability of findings to advanced disease [12]. Wattana et al. similarly found that a substantial proportion of emergency physicians reported knowledge gaps related to cancer therapeutics and serious cancer-related complications [17]. These gaps persist despite strategic priorities from professional bodies, such as the Oncology Nursing Society 2024–2027 research agenda, which calls for strengthening oncology nurses’ roles in symptom management, communication, and early palliative care integration [18]. To the best of our knowledge, no prior scoping review has specifically mapped nursing roles, including nurse-led interventions, nursing competencies, telehealth nursing, and caregiver-directed nursing practice in the early integration of palliative and supportive care for adults with advanced cancer. This review addresses that gap.

The conceptual focus of this review centers on nursing roles within early palliative and supportive care integration, specifically, how nurses contribute to symptom assessment and management, advance care planning, telehealth and telephone follow-up, care coordination, and caregiver support. While nursing competencies and educational context are addressed, these are discussed in relation to their clinical implications for patient and caregiver outcomes, not as ends in themselves. This review is intended to be directly informative for clinical oncology practice, healthcare system design, and nursing policy.

A scoping review is the most appropriate methodology to address this gap because the breadth and heterogeneity of nursing roles in early palliative care integration across settings, populations, and study designs are not yet sufficiently mapped to support a systematic review or meta-analysis. By mapping the available evidence, this review aims to identify what is known, clarify how nursing contributes to the integration of early palliative care, and highlight gaps that should inform future research, practice, and policy.

## 2. Materials and Methods

### 2.1. Study Design

This study was conducted as a scoping review to map the breadth and nature of evidence on nursing roles in the early integration of palliative and supportive care for adults with advanced cancer. The review followed the five-stage methodological framework proposed by Hilary Arksey and Lisa O’Malley [19], which includes: (1) identifying the research question; (2) identifying relevant studies; (3) study selection; (4) charting the data; and (5) collating, summarizing, and reporting the results. Reporting adhered to the Preferred Reporting Items For Systematic Reviews and Meta-Analyses (PRISMA-ScR) guideline.

### 2.2. Conceptual Focus and Eligibility Criteria

We used a Population, Concept, Context (PCC) approach to define eligibility, with the detaild inclusion and exclusion criteria summarized in Table 1 [20].

Studies were included if they: (1) examined nursing roles, nurse-led interventions, or nursing-relevant components of early palliative and/or supportive care in oncology, (2) involved adult patients with cancer and/or their caregivers, (3) reported at least one patient-, caregiver-, or system-level outcome (e.g., symptom burden, quality of life, advance care planning, satisfaction, communication, or service utilization), (4) were published in peer-reviewed journals in English between January 2016 and November 2025; and used any empirical or evidence-synthesizing design, including randomized or quasi-experimental studies, observational cohort or cross-sectional studies, qualitative studies, mixed-methods studies, narrative reviews, or descriptive models of care. Evidence syntheses (systematic reviews and meta-analyses) were included to ensure comprehensive mapping of the existing knowledge base.

Evidence syntheses (systematic reviews, meta-analyses, and narrative reviews) were included even when nursing roles were not the primary focus, provided the review addressed nursing-relevant components of early palliative care or reported outcomes applicable to nursing practice. This inclusive approach is consistent with the mapping purpose of scoping reviews and ensures that the broader context within which nursing roles operate is captured.

We excluded studies that focused exclusively on pediatric populations; did not address nursing roles or did not include a nursing-relevant component; examined palliative care only at the very end of life without an early integration component; were conference abstracts, protocols without results, editorials, commentaries, or non-peer-reviewed reports; or were published in languages other than English.

A comprehensive search was conducted across four electronic databases: PubMed, CINAHL, Scopus, and ScienceDirect, covering the period from January 2016 to November 2025. These databases were selected because they provide comprehensive coverage of biomedical, nursing, and multidisciplinary health literature. The search strategy was developed iteratively by the research team and combined controlled vocabulary and free text terms related to cancer, palliative and supportive care, and nursing. A typical search string included combinations of terms such as: (“cancer” OR “oncology”) AND (“palliative care” OR “supportive care” OR “early palliative care”) AND (“nurse-led” OR “nursing role” OR “nursing care” OR “advance care planning”). Search strategies were tailored to each database, and the complete strings and development process are provided in Supplementary Tables S1 and S2.

To ensure comprehensive coverage, we also conducted backward and forward citation tracking of all included studies and relevant systematic reviews using Google Scholar and Web of Science. Reference lists of included articles were hand-searched to identify additional eligible publications. In line with PRISMA-ScR guidance, the review methods were defined a priori. The registration in Open Science Framework is DOI: 10.17605/OSF.IO/2NYP4.

### 2.3. Study Selection

**Figure 1:**
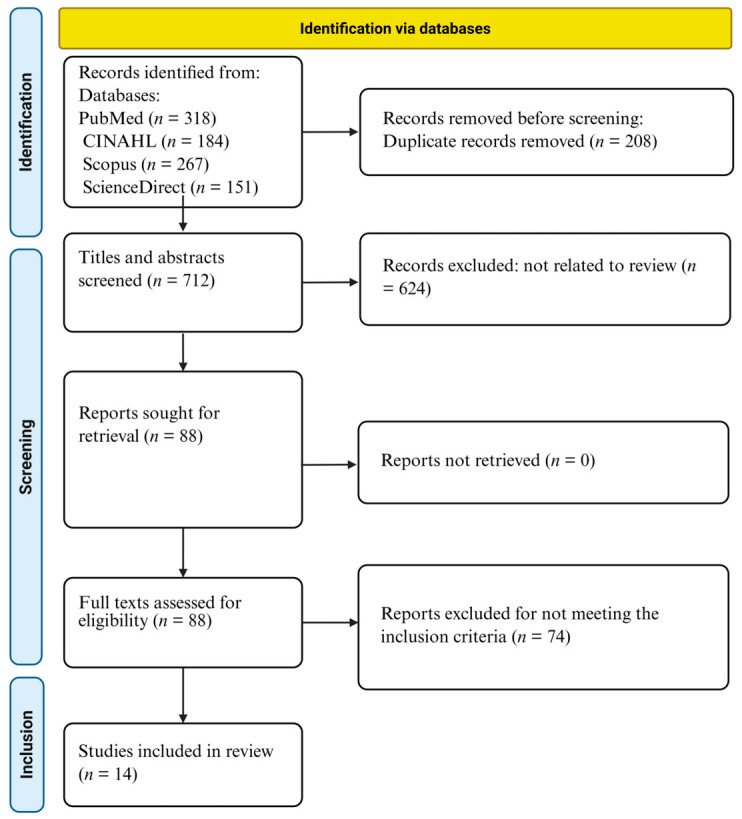
Preferred Reporting Items for Systematic Reviews and Meta-Analyses extension for Scoping Reviews (PRISMA-ScR) flow diagram of the study selection process. The database search yielded 920 records (PubMed (*n* = 318); CINAHL (*n* = 184); Scopus (*n* = 267); ScienceDirect (*n* = 151). After removal of 208 duplicates, 712 titles and abstracts were screened, resulting in 88 full-text articles assessed for eligibility. Fourteen studies met the inclusion criteria and were included in the final synthesis.

All records identified from the database searches were imported into a reference management program (EndNote software, V21), and duplicates were removed. Two independent reviewers (O.A. and P.T.) screened titles and abstracts, followed by a full-text review of potentially eligible articles. Discrepancies were resolved through discussion with the third author (S.A). The selection process is summarized in the PRISMA-ScR flow diagram (Figure 1).

### 2.4. Data Charting

A standardized data-charting form was developed, piloted on a subset of studies, and subsequently refined by the review team. For each included study, we extracted the following: author and year, country, study design, setting, sample characteristics, cancer type and stage (when reported), description of the nursing role or intervention (including early palliative and supportive care components), outcomes measured (patient, caregiver, or system level), and key findings. One reviewer performed the initial data extraction, and a second reviewer verified all entries for accuracy and completeness.

The 14 included studies encompassed randomized controlled trials, quasi-experimental studies, retrospective cohort and observational studies, a multicenter cross-sectional survey, a qualitative interpretive descriptive study, a meta-analysis, systematic and narrative reviews, and a descriptive model of care. Settings spanned emergency departments, oncology and palliative care units, surgical oncology clinics, community and home-based programs, and telehealth platforms across the United States, Canada, China, Italy, Egypt, Romania, and the Netherlands.

### 2.5. Synthesis of Results

Consistent with scoping review methodology, we did not perform a formal risk-of-bias assessment, as the aim was to map the extent and nature of the evidence rather than to generate pooled effect estimates. Instead, we conducted an inductive thematic synthesis across the charted data. Studies were grouped according to the primary focus of the nursing role or intervention (e.g., early palliative care integration, advanced care planning, telephone-based symptom management, nursing competencies, models of care, and caregiver outcomes). Through iterative team discussion, we identified six analytical domains and summarized how different study types contributed to each domain. A summary table (Table 2) describes the characteristics of the included studies, and a thematic synthesis table (Table 3) outlines the domains, outcome measures, and principal findings. Consistent with the PRISMA-ScR guidelines for scoping reviews, a formal assessment of methodological quality (risk of bias) of the included studies was not performed. The purpose of this review was to map the breadth of available evidence rather than to assess the quality of individual studies for meta-analysis. Data were synthesized descriptively to categorize interventions and summarize their impact on patient outcomes.

The inclusion of evidence syntheses (systematic reviews, meta-analyses, and narrative reviews) alongside primary data across sources. In line with scoping review methodology, we did not attempt to de-duplicate primary studies underlying each synthesis. Instead, the included reviews and meta-analyses were treated as independent evidence sources representing the synthesized knowledge on topics. The findings from evidence syntheses were not pooled with primary study data; rather, each was summarized at the review level. Where findings from evidence syntheses appeared consistent or inconsistent with primary studies, we noted this in our narrative synthesis. This approach is standard practice for scoping reviews and does not affect the validity of the mapping exercise.

## 3. Results

### 3.1. Study Selection and Characteristics

The database search yielded 920 records (PubMed, 318, CINAHL: 184, Scopus: 267, ScienceDirect: 151). After removing 208 duplicates, 712 titles and abstracts were screened, resulting in 88 full-text articles assessed for eligibility. A final total of 14 studies met the inclusion criteria (Figure 1). The included studies comprised randomized controlled trials (*n* = 3), a quasi-experimental study (*n* = 1), a multicenter cross-sectional study (*n* = 1), a qualitative study (*n* = 1), retrospective cohort studies (*n* = 2), systematic reviews (*n* = 2), narrative reviews (*n* = 2), a meta-analysis (*n* = 1), and a descriptive model of care (*n* = 1). Studies were conducted across multiple countries (the United States, Canada, China, Italy, Egypt, Romania, and the Netherlands) and healthcare settings (emergency departments, oncology wards, palliative care units, surgical oncology clinics, community-based settings, and telehealth platforms). Sample sizes of primary research ranged from 19 (qualitative interviews) to 1198 (multicenter survey).

Thematic analysis of the 14 included studies identified six analytical domains: (1) early palliative care integration and patient outcomes, (2) nurse-led advance care planning interventions, (3) telephone-based symptom management, (4) nursing competencies and practice gaps, (5) conceptual frameworks and models of care, and (6) caregiver outcomes. Themes were identified through repetitive comparison of extracted data across studies, centering on the primary nursing role or intervention described and the outcomes reported.

**Figure 2:**
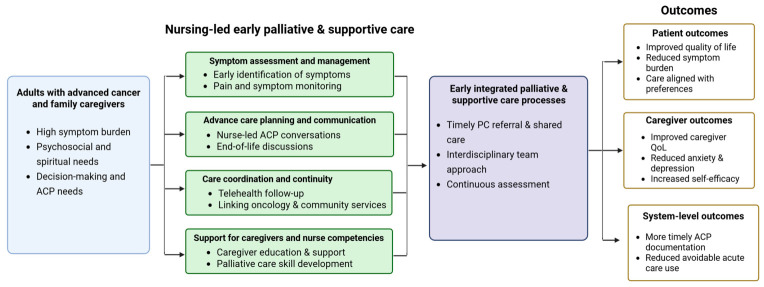
Conceptual model of nursing-led early palliative and supportive care for adults with advanced cancer, based on thematic synthesis of 14 included studies. The figure illustrates the nursing role clusters and associated outcomes identified in the scoping review literature. This model is derived from a descriptive mapping of available evidence and is intended to serve as a conceptual organizing framework rather than a prescriptive or validated care pathway. ACP: advance care planning; PC: palliative care; QoL: quality of life.

A conceptual overview of these domains and nursing roles in early palliative and supportive care is presented in Figure 2. Table 3 provides a detailed synthesis of these domains with corresponding outcome measures and contributing studies.

### 3.2. Early Palliative Care Integration and Patient Outcomes

Four studies examined the impact of early palliative care (EPC) integration on patient clinical outcomes. Creangă-Murariu et al., in a systematic review of 41 RCTs, found that 32 trials (78%) demonstrated significant clinical benefit, with EPC consistently improving quality of life (QOL) and reducing symptom burden; some trials also suggested slowed disease progression, although substantial heterogeneity in timing, frequency, and integration approaches was noted across studies [29]. Grudzen et al. in a single-blind RCT of 136 patients with advanced cancer presenting to the emergency department, reported that ED-initiated palliative care significantly improved QOL at 12 weeks (mean Functional Assessment of Cancer Therapy—General (FACT-G) increase: 5.91 ± 16.65 vs. 1.08 ± 16.00; *p* = 0.03), with longer median survival in the intervention group (289 days; 95% CI: 128–453) compared with usual care (132 days; 95% CI: 80–302), though this did not reach statistical significance (*p* = 0.20) [27]. Abdel-Aziz et al., using a quasi-experimental design with 140 patients in Egypt, demonstrated significant reductions in symptom severity: physical symptoms (mean: 3.5 ± 1.2 to 1.0 ± 0.5), psychological symptoms (5.3 ± 1.8 to 2.5 ± 1.0), and emotional/spiritual needs (4.0 ± 1.3 to 1.5 ± 0.8), all *p* < 0.001 [33]. Physical functioning improved from 60.0 to 80.0 and emotional functioning from 55.0 to 75.0 on the EORTC QLQ-C30 (*p* < 0.001). Vanbutsele et al., in a Belgian randomized controlled trial of 186 patients with incurable solid tumors and a prognosis of one year or less, found that early and systematic integration of palliative care delivered primarily by a specialized palliative care nurse with physician backup for complex situations significantly improved quality of life at three months compared to standard multidisciplinary care (*p* = 0.02) [34]. The trial is notable for its explicit nurse-led delivery model, which demonstrates that nurses can serve as the primary providers of structured early palliative care integration in oncology settings.

### 3.3. Nurse-Led Advance Care Planning Interventions

Two studies evaluated the effectiveness of nurse-led interventions on advance care planning (ACP) uptake. Cohen et al. in a secondary analysis of a cluster RCT of 672 patients, found that nurse-led primary palliative care significantly increased end-of-life conversations (EOLC: 45.1% vs. 14.8%; adjusted OR = 5.28, 95% CI: 3.10–8.97, *p* < 0.001) and advance directive (AD) completion (43.2% vs. 18.1%; adjusted OR = 3.68, 95% CI: 1.89–7.16, *p* < 0.001) [24]. Bansal et al., in a retrospective analysis of 326 surgical oncology patients, reported that AD designation increased from 72.0% to 85.4% following workflow integration (*p* = 0.004), with palliative care consultation being the strongest predictor (OR = 41.48, 95% CI: 9.59–179.43, *p* < 0.001) [21]. Racial and ethnic disparities were also observed, with patients self-identifying as a race other than non-Hispanic White being less likely to designate ADs (OR = 0.36, *p* = 0.008).

### 3.4. Telephone-Based Symptom Management

Valenti et al., in a retrospective observational study of 171 patients and 323 phone calls, demonstrated the feasibility of nurse-led telephone follow-up within an early palliative care program [26]. The most frequent reasons for calls were pain management (38.4%), clinical updates (23.8%), medication management (18.9%), and scheduling (18.3%). Of 323 calls, 210 (65%) were managed by the nursing case manager alone, with an overall effectiveness rate of 87.6% based on symptom resolution or stabilization within 7 days.

### 3.5. Nursing Competencies and Practice Gaps

Two studies assessed nursing competencies relevant to palliative care delivery. Nie et al. (2025), in a multicenter cross-sectional study of 1198 oncology nurses across 26 hospitals in China, reported a total PCPS score of 67.17 ± 12.57, with physical symptom care scoring highest (32.50 ± 6.10) and communication scoring lowest (11.58 ± 2.48) [22]. Palliative care practice ability was strongly correlated with overall nursing competence (r = 0.77, *p* < 0.01) and pain assessment ability (r = 0.56, *p* < 0.01); multiple regression identified being female, higher education, interest in palliative care, core competence, and pain assessment as significant predictors (Adjusted R^2^ = 0.668, *p* < 0.05). Kilgour et al., in a qualitative study of 19 oncology nurses, identified three themes: (1) uncertainties related to the nursing role in ACP, (2) educational and training gaps, and (3) structural barriers including lack of time, space, privacy, and interdisciplinary team dynamics [25].

### 3.6. Conceptual Frameworks and Models of Care

Four studies proposed conceptual frameworks or clinical models to guide early palliative care integration (Table 2). Hui and Bruera articulated a conceptual framework emphasizing team-based, timely, and targeted palliative care: an interdisciplinary approach addresses multidimensional care needs, timely care serves as preventive care, and targeted care identifies patients most likely to benefit from specialist palliative care [28]. Koekkoek et al., synthesizing 140 articles, proposed a comprehensive five-phase palliative and supportive care framework for high-grade glioma patients and their caregivers, recommending that EPC interventions and ACP be proactively addressed in each disease phase [31]. Shaulov et al. proposed a three-level care model (primary/community, secondary/hospital specialists, tertiary/specialized palliative care consultants) for hematological malignancies, noting comparable symptom burdens to solid tumors but unique barriers to early palliative care access [30]. Lelond and Kim described a clinical nurse specialist (CNS)-led EPC model in Manitoba, Canada, receiving 269 referrals with 235 consults completed in 18 months; 157 (66.8%) were for symptom management, and 124 (52.8%) were seen before biopsy confirmation, demonstrating the feasibility of nurse-led EPC during the diagnostic phase [32].

### 3.7. Caregiver Outcomes

Chow et al., in a meta-analysis of 49 trials involving 8554 caregivers of patients with advanced cancer, reported significant improvements in overall QoL (SMD = 0.24, 95% CI: 0.10–0.39), mental well-being (SMD = 0.14), anxiety (SMD = 0.27), and depression (SMD = 0.34, *p* < 0.001) [23]. Narrative synthesis of additional studies also demonstrated improvements in caregiver self-efficacy and bereavement outcomes. Although the Chow et al. meta-analysis was not restricted to nurse-led trials, nurses were involved in delivering caregiver-targeted interventions across many of the contributing studies; its inclusion maps the evidence base for caregiver-directed care that nurses are well-positioned to provide in clinical practice.

## 4. Discussion

Importantly, this review is the first scoping review to map nursing-specific contributions to early palliative care integration across diverse healthcare settings and study designs, providing a synthesis that existing systematic reviews, which focus on overall EPC efficacy rather than nursing roles specifically, have not previously offered. The 14 studies are on adults with advanced cancer. Overall, the findings indicate that when nurses are actively involved in early palliative and supportive care through baseline assessment, advance care planning, telephone follow-up, community-based models, and specialist roles, patient and caregiver outcomes improve, and care processes become more aligned with patient needs. A key theme was the consistent association between early palliative care integration and improved patient outcomes. The systematic review by Creangă-Murariu et al. reported that 32 of 41 randomized trials (78%) demonstrated significant clinical benefit of early palliative care, including improved quality of life and reduced symptom burden in advanced cancer [29]. Individual intervention studies, such as Grudzen et al.’s [27] emergency department-initiated palliative care trial and Abdel-Aziz et al.’s [33] nurse-led community program, similarly showed clinically meaningful reductions in symptom severity and better functional outcomes for patients with advanced malignancies. Together, these data reinforce international recommendations that palliative care should be introduced early in the metastatic disease trajectory rather than reserved for the end of life, and they highlight that nurses are often the professionals operationalizing these early interventions in practice.

Nurse-led advanced care planning (ACP) emerged as a particularly impactful domain. In the CONNECT trial [29] analysis, nurse-led primary palliative care within community cancer centers substantially increased patient-reported end-of-life conversations and advance directive completion compared with standard oncology care. Similarly, Bansal et al. [21] found that integrating palliative care and ACP prompts into the surgical oncology workflow increased advance directive designation among patients with advanced abdominal and soft-tissue malignancies, with palliative care consultation showing a very strong association with documentation. However, Bansal et al. also reported racial and ethnic disparities in advance directive designation, underscoring the need for nurse-led ACP interventions that are explicitly designed to address equity and cultural safety [26].

Nurse-led telephone and telehealth symptom management offers another important avenue for expanding early palliative and supportive care. Valenti et al. [26] demonstrated that a nurse-led telephone follow-up service within an early palliative care program was highly utilized, with most calls related to pain, clinical updates, and medication management, and nearly two-thirds of contacts effectively managed by nurses alone. These findings suggest that nurses can provide timely, low-threshold support between in-person visits, potentially reducing unnecessary acute care utilization and improving continuity of care for patients and families. Telehealth-based nursing interventions may be especially valuable for patients who face geographic or functional barriers to accessing specialized palliative services.

The findings converge on several key themes: the clinical benefit of early palliative care integration, the effectiveness of nurse-led interventions in advanced directive planning and symptom management, persistent competency and structural gaps in nursing practice, and the emerging importance of caregiver-inclusive approaches. These themes are discussed below in relation to the broader literature.

At the same time, the review reveals important gaps in nursing competencies and practice environments. In a large multicenter survey from China, Nie et al. reported moderate overall palliative care practice scores among oncology nurses, with the lowest performance in communication, despite strong associations between core competence, pain assessment ability, and overall practice [22]. Complementing these quantitative findings, Kilgour et al. found that oncology nurses in Canada experienced uncertainty about their role in ACP, perceived educational and training gaps, and encountered structural barriers such as limited time, privacy, and inconsistent team support for nurse-led ACP [25]. These studies collectively suggest that strengthening early palliative and supportive care requires not only knowledge-based education in symptom management and ACP but also investment in advanced communication training, clear role delineation, and organizational changes that enable nurses to engage in complex conversations and longitudinal relationships with patients.

Conceptual frameworks and models of care included in this review further clarify how nursing roles can be embedded across the trajectory of advanced cancer. Hui et al. [28] state-of-the-science review proposes a team-based, timely, and targeted approach to palliative care that emphasizes interdisciplinary collaboration and proactive identification of patients most likely to benefit from specialist input. Shaulov et al. describe a three-level model (primary, secondary, and tertiary palliative care) for hematological malignancies, in which oncology and hematology clinicians support secondary palliative care and specialist teams manage more complex needs, a structure that implicitly relies on nurses at all levels [30]. Koekkoek et al. [31] presented a five-phase palliative and supportive care framework for high-grade glioma that integrates early palliative care and ACP throughout the disease course and highlights the importance of continuous support for both patients and caregivers. Lelond and Kim’s [32] descriptive model of a clinical nurse specialist-led early palliative care service for high-risk hepato-pancreato-biliary cancers further demonstrates that nurses can feasibly assess symptoms, goals of care, and referral needs, often even before histological confirmation, thereby embedding early palliative principles at the diagnostic stage.

Caregiver-focused and dyadic interventions represent an additional, often under-recognized dimension of early palliative and supportive care. In a large meta-analysis, Chow et al. showed that supportive interventions targeting caregivers and patient–caregiver dyads improved caregiver quality of life, anxiety, depression, and self-efficacy in the context of advanced cancer [23]. These findings are highly relevant to nursing practice, as nurses are frequently the first to recognize caregiver distress and can play a central role in referring caregivers to supportive programs or delivering structured psychoeducational and supportive interventions themselves.

Taken together, these findings have several *implications* for practice, education, and policy. First, based on the convergent findings of this review, oncology, emergency, and community services may consider prioritizing early, nurse-led palliative and supportive care interventions that combine systematic symptom assessment, ACP, caregiver support, and care coordination. The evidence mapped in this review is consistent with but does not yet definitively establish a causal benefit of nurse-specific involvement as distinct from broader interdisciplinary early palliative care integration. Rigorous evaluation of nurse-specific models through randomized and implementation studies is needed before definitive practice recommendations can be made. Second, nursing education at undergraduate and postgraduate levels, as well as continuing professional development, should incorporate structured training in palliative care communication, culturally responsive ACP, and longitudinal symptom management, building on the competence domains identified in survey and qualitative studies. Third, organizational leaders should redesign workflows and staffing models to protect time for nurse-led ACP and complex symptom discussions, support clinical nurse specialist or nurse navigator roles, and integrate telehealth as a standard modality for early supportive and palliative care delivery.

This review has limitations inherent to scoping methodology. As recommended for scoping reviews following the Arksey and O’Malley framework, we did not perform a formal risk-of-bias assessment or meta-analysis, and the included studies were heterogeneous in design, populations, settings, and outcomes, limiting the ability to directly compare effects across interventions [19]. The inclusion of evidence syntheses (two systematic reviews, two narrative reviews, and one meta-analysis) alongside primary studies introduces the possibility of overlapping primary data; however, consistent with scoping review methodology, each synthesis was treated as an independent evidence source and was summarized at the review level rather than being pooled with primary studies. In cases where findings appeared to conflict across study types (e.g., heterogeneous effects of early palliative care timing reported by the systematic review by Creangă-Murariu et al. [29] versus the more specific findings from primary trials), we acknowledged this heterogeneity in the narrative synthesis rather than attempting to resolve it statistically. Most studies were conducted in high-income countries, potentially limiting the generalizability of findings to low- and middle-income settings, although the inclusion of nurse-led community palliative programs from such contexts is encouraging. Furthermore, some domains such as emergency department-initiated palliative care, hematological malignancies, and culturally tailored ACP are represented by relatively few studies and warrant further investigation. Three included studies require specific acknowledgment regarding eligibility boundaries. The cross-sectional survey by Nie et al. maps nursing competencies rather than patient outcomes and was included under the ‘nursing competencies and practice gaps’ domain to characterize the nursing work relevant to early palliative care delivery [22]. The meta-analysis by Chow et al. addresses caregiver outcomes broadly and does not restrict to nurse-led interventions; it was included to map the evidence base for caregiver-directed care applicable to nursing roles [23]. The quasi-experimental study by Abdel-Aziz et al. enrolled patients with cancer and other life-limiting illnesses rather than exclusively advanced cancer; findings were nonetheless included as they reflect nurse-led community palliative care relevant to the broader population [33]. These inclusions reflect the mapping rather than the evaluative purpose of scoping reviews and are acknowledged in interpreting our findings.

Despite these limitations, this scoping review provides a comprehensive overview of how nurses currently contribute to the early integration of palliative and supportive care for adults with advanced cancer. Future research should include rigorous evaluations of nurse-led models in diverse health systems, including randomized and implementation studies, and should focus on strategies to overcome communication, role, and structural barriers identified in existing work.

## 5. Conclusions

Nurses occupy a pivotal position in operationalizing early palliative and supportive care for adults with advanced cancer. Evidence from diverse settings indicates that nurse-led and nursing-relevant interventions can improve patient and caregiver outcomes, enhance ACP uptake, and support continuity of symptom management across the cancer trajectory. Expanding structured, nurse-led early palliative and supportive care models, coupled with targeted education and organizational reform, is essential to optimize patient-centered outcomes and to ensure that patients with advanced cancer receive timely, person-centered palliative and supportive care.

## Figures and Tables

**Table 1:** Population–Concept–Context (PCC) framework for eligibility criteria.

Conceptual Name	Definition
Population	Adults (≥18 years) diagnosed with cancer, with particular focus on studies involving advanced, metastatic, or incurable disease.
Concept	Early integration of palliative and/or supportive care, with an explicit nursing component. This included nurse-led or nursing-relevant interventions, roles, competencies, or models that addressed early symptom assessment and management, advance care planning, psychosocial or spiritual support, care coordination, or caregiver support within a palliative or supportive care framework.
Context	Any healthcare setting where early palliative or supportive care was delivered, including emergency departments, oncology inpatient units and outpatient clinics, palliative care units, surgical oncology services, community-based and home-care services, and telehealth or telephone follow-up programs.

**Table 2:** Summary of 14 studies included in this review.

Author	Country	Study Design	Sample Size	Cancer Type	Nursing Role/Component	Main Findings
Bansal (2023) [21]	USA	Retrospective	326 patients	Advanced abdominal and soft tissue malignancies	Palliative care nurse involvement in workflow integration; surgical oncology nursing care coordination	AD designation increased from 72.0% to 85.4% (*p* = 0.004). AD designation associated with palliative care consultation (OR 41.48, *p* < 0.001) and workflow integration (OR 2.05, *p* = 0.048). Documentation rates did not significantly increase (48.9% vs. 56.3%, *p* = 0.19).
Nie (2025) [22]	China	Multicenter cross-sectional study	1198 nurses	Oncology department	Direct nursing role: assessment of palliative care practice ability, competencies, communication skills	Total PCPS score: 67.17 ± 12.57. Positive correlations between practice ability and core competence (r = 0.77, *p* < 0.01) and pain assessment ability (r = 0.56, *p* < 0.01). Female gender, higher education, interest in palliative care, pain assessment, and core competence were positive predictors (Adjusted R^2^ = 0.668, *p* < 0.05).
Chow (2023) [23]	Canada	Meta-analysis	8554 caregiver (49 trials)	Advanced cancer	Nurses involved in delivering caregiver-targeted psychoeducational and supportive interventions across contributing trials	Significant improvements in overall QoL (SMD = 0.24, 95% CI 0.10–0.39), mental well-being (SMD = 0.14), anxiety (SMD = 0.27), and depression (SMD = 0.34, *p* < 0.001). Narrative synthesis showed improvements in self-efficacy and grief. Included to map caregiver-targeted evidence relevant to nursing practice; many contributing trials involved nurse-delivered or nurse-co-delivered supportive interventions for caregivers.
Cohen (2023) [24]	USA	Secondary analysis of cluster RCT	672 patients	Advanced cancer	Nurse-led primary palliative care delivered within community cancer centers	EOLC: 45.1% intervention vs. 14.8% control (adjusted OR 5.28, 95% CI 3.10–8.97, *p* < 0.001). AD completion: 43.2% intervention vs. 18.1% control (adjusted OR 3.68, 95% CI 1.89–7.16, *p* < 0.001).
Kilgour (2025) [25]	Canada	Qualitative interpretive descriptive study	19 oncology nurses	Various oncology settings	Oncology nurses leading or participating in advance care planning conversations	Identified three themes: (1) uncertainties related to the nursing role in ACP, (2) educational and training environment gaps, and (3) structural barriers, including lack of time, space, privacy, and team dynamics affecting interdisciplinary collaboration.
Valenti (2023) [26]	Italy	Observational retrospective study	171 patients	Advanced cancer (early palliative care)	Nurse-led telephone follow-up as primary contact point; nursing case manager managing calls independently	323 total phone calls. Most common requests: pain management (38.4%), clinical updates (23.8%), medication management (18.9%), and scheduling (18.3%). 87.6% effectiveness in telephone management. 210/323 calls handled by a nurse alone.
Grudzen (2016) [27]	USA	Single blind RCT	136 patients	Advanced cancer presenting to emergency department	Palliative care team including nurse practitioner and social worker; nurse assessment on ED presentation	QoL improved significantly in the intervention group (mean increase 5.91 vs. 1.08, *p* = 0.03). Median survival: 289 days intervention vs. 132 days control (*p* = 0.20, not significant). No significant differences in depression or ICU admission.
Hui (2018) [28]	USA/Canada	Narrative review	N/A (review articles)	Advanced cancer	Nurses as integral members of team-based palliative care; APN-delivered components; nursing within interdisciplinary model	Proposed conceptual frameworks for integrating palliative care: a team-based approach addresses multidimensional needs, timely care is preventive, and targeted care identifies patients most likely to benefit. Emphasized the need for personalized palliative care delivery.
Creangă-Murariu (2025) [29]	Romania	Systematic review	14 RCTs	Advanced, incurable or metastatic cancer	APNs and RNs co-delivered EPC in multiple contributing trials (e.g., ENABLE trials, Temel trials)	32/41 trials demonstrated significant clinical benefit. EPC consistently improved QOL and reduced symptom burden. Nursing professionals (APNs, RNs, nurse practitioners) delivered or co-delivered EPC in a substantial proportion of the included trials; the review was included to map the broader EPC evidence base within which nursing roles operate, in line with the PCC framework’s ‘nursing-relevant components’ criterion.
Shaulov (2022) [30]	Canada	Narrative review	N/A (review articles)	Hematological malignancies	Nursing at primary and secondary levels of three-tier PC model; nurse-led symptom management in haemato-oncology units	Patients with hematological malignancies have a comparable symptom burden to solid tumors but face barriers to early palliative care. Proposed three-level care model: primary (community), secondary (hospital specialists), tertiary (specialized PC consultants). Emphasized the need for early integration despite prognostic uncertainty.
Koekkoek (2023) [31]	The Netherlands, Italy, Switzerland, Canada, USA, India, UK, Australia (international panel)	Systematic review	140 articles included (search yielded 5262 articles)	Adults with malignant brain tumors (gliomas and brain metastases)	Nursing program (cognitive behavioral interventions) reduced fatigue/anxiety; nursing within five-phase palliative framework	A comprehensive framework of palliative and supportive care for high-grade glioma patients and caregivers was proposed with five disease trajectory phases. No pharmacological agent significantly improved fatigue or neurocognition in RCTs. Early palliative care interventions and advanced care planning should be part of each disease phase.
Lelond & Kim (2025) [32]	Canada (Manitoba)	Descriptive model care (with implementation data)	269 referrals received; 235 consults completed in 18 months	Advanced pancreatic cancer, cholangiocarcinoma, and hepatocellular carcinoma	Clinical nurse specialist (CNS)-led model; CNS conducts symptom assessment, goals of care, and referral—often pre-biopsy	Of 235 consults, 157 were for symptom management and 61 for goals of care discussion. 124 patients were seen before biopsy confirmation. At initial consult: 100 had goals aligned with chemotherapy, 90 with palliative care alone, and 45 were unsure. 136 went on to see the medical oncologist; 76 received chemotherapy. Key barriers: lack of understanding of CNS role, confusion between EPC and end-of-life care, limited resources. Facilitators: stakeholder buy-in, alignment with national palliative care frameworks, leveraging existing referral systems.
Abdel-Aziz, Zaghamir, & Ibrahim (2025) [33]	Egypt	Quasi-experimental design (pre- and post-test)	140 adult patients	Cancer and other life-limiting illnesses	Structured nurse-led community palliative care program; nurses delivering holistic assessment and care	Significant reduction in symptom severity: physical symptoms (mean score 3.5 → 1.0), psychological symptoms (5.3 → 2.5), and emotional and spiritual needs (4.0 → 1.5), all *p* < 0.001. EORTC QLQ-C30 showed improved QoL: physical functioning (60.0 → 80.0) and emotional functioning (55.0 → 75.0). Participants reported improved perceptions of social support and general well-being. Highlights the feasibility and positive impact of structured nurse-led community palliative care in a low- and middle-income country setting.
Vanbutsele et al. (2018) [34]	Belgium	RCT	186 patients	Incurable solid tumors (prognosis ≤ 1 year)	Specialized palliative care nurse as primary care provider; physician referral only for complex cases	Early and systematic palliative care integration, delivered by a specialized palliative care nurse with physician backup, significantly improved QoL at 3 months (*p* = 0.02) compared to standard multidisciplinary care. No significant differences in survival, anxiety, depression, or symptom scores. Nursing role was explicit: the intervention was nurse-led with physician referral for complex cases.

**ACP**: advance care planning; **AD**: advance directive; **APN**: advanced practice nurse; **ASD**: Antiseizure drug; **CCMB**: cancer care Manitoba; **CI**: confidence interval; **CNS**: clinical nurse specialist; **EANO**: European Association of Neuro-Oncology; **ED**: emergency department; **EHR**: electronic health records, **ENABLE**: Educate, Nurture, Advise, Before Life Ends; **EOLC**: end-of-life conversation; **EORTCQLQ-C30**: European organization for research and treatment of cancer quality of life questionnaire; **EPC**: early palliative care; **EPCS**: end-of-life professional caregiver survey; **ESAS**: Edmonton symptom assessment scale; **FACT-G**: functional assessment of cancer therapy general measure; **ICU**: intensive care unit; **N/A**: not available; **OR**: odds ratio; **PC**: palliative care; **PCC**: Population, Concept, Context; **PCPS**: palliative care self-reports practices scale; **PHQ-9**: patients health questionnaire-9; **POS**: palliative outcome scale; **QoL**: quality of life; **RCT**: randomized control trial; **RN,** registered nurse; **Self-PAC**: self-perceived pain assessment knowledge and confidence scale; **SMD**: Standardized mean difference.

**Table 3:** Synthesis of Key Findings Across Included Studies (*n* = 14).

Analytical Domain	Author (Year)	Study Design	Key Outcome Measures	Principal Findings
EPC and Patient Outcomes	Creangă-Murariu et al. (2025) [29]	Systematic review (41 RCTs)	QoL, symptom burden, disease progression	32/41 (78%) trials showed significant clinical benefit; EPC improved QoL and reduced symptom burden
	Grudzen et al. (2016) [27]	Single-blind RCT (*n* = 136)	FACT-G, survival, depression	QoL mean increase of 5.91 vs. 1.08 (*p* = 0.03); median survival 289 vs. 132 days (*p* = 0.20)
	Abdel-Aziz et al. (2025) [33]	Quasi-experimental (*n* = 140)	POS, ESAS, EORTC QLQ-C30	Physical symptoms 3.5 → 1.0; emotional/spiritual 4.0 → 1.5; physical functioning 60.0 → 80.0 (all *p* < 0.001)
	Vanbutsele et al. (2018) [34]	RCT (*n* = 186)	QoL (EORTC QLQ-C30)	QoL significantly improved at 3 months (*p* = 0.02); the nurse-led model demonstrated the feasibility of CNS as the primary EPC provider
Nurse-Led ACP Interventions	Cohen et al. (2023) [24]	Secondary analysis of cluster RCT (*n* = 672)	EOLC, AD completion	EOLC: 45.1% vs. 14.8% (adj. OR = 5.28, *p* < 0.001); AD: 43.2% vs. 18.1% (adj. OR = 3.68, *p* < 0.001)
	Bansal et al. (2023) [21]	Retrospective cohort (*n* = 326)	AD designation, AD documentation	AD designation 72.0% → 85.4% (*p* = 0.004); PC consultation OR = 41.48 (*p* < 0.001)
Telephone Symptom Management	Valenti et al. (2023) [26]	Retrospective observational (*n* = 171)	Call reasons, nurse management rate, effectiveness	87.6% effectiveness; pain (38.4%) most common; 65% calls managed by nurse alone
Nursing Competencies and Gaps	Nie et al. (2025) [22]	Multicenter cross-sectional (*n* = 1198)	PCPS, EPCS, Self-PAC	PCPS = 67.17 ± 12.57; communication lowest; core competence r = 0.77; Adj. R^2^ = 0.668
	Kilgour et al. (2025) [25]	Qualitative interpretive descriptive (*n* = 19)	Thematic analysis	Three barriers: role uncertainty, training gaps, structural barriers (time, space, privacy)
Frameworks and Models	Hui & Bruera (2018) [28]	Narrative review	Conceptual framework	Team-based, timely, targeted palliative care; interdisciplinary approach; personalized delivery
	Koekkoek et al. (2023) [31]	Systematic review (140 articles)	Framework development	Five-phase palliative care framework for glioma; ACP across all disease phases
	Shaulov et al. (2022) [30]	Narrative review	Model of care	Three-level model (primary, secondary, tertiary) for hematological malignancies
	Lelond & Kim (2025) [32]	Descriptive model of care (*n* = 235 consults)	Referral data, consult outcomes	269 referrals; 157 for symptom management; 124 seen pre-biopsy; CNS-led feasibility demonstrated
Caregiver Outcomes	Chow et al. (2023) [23]	Meta-analysis (49 trials, *n* = 8554)	QoL, anxiety, depression, self-efficacy	QOL SMD = 0.24; depression SMD = 0.34; anxiety SMD = 0.27 (*p* < 0.001); improved self-efficacy and grief

**ACP**: advance care planning; **AD**: advance directive; adj. **OR**: adjusted odds ratio; **CNS**: clinical nurse specialist; **EOLC**: end-of-life conversation; **EORTC QLQ-C30**: European Organization for Research and Treatment of Cancer Quality of Life Questionnaire; **EPC**: early palliative care; **EPCS**: End-of-Life Professional Caregiver Survey; **ESAS**: Edmonton Symptom Assessment Scale; **FACT-G**: Functional Assessment of Cancer Therapy—General; **PCPS**: Palliative Care Self-Report Practice Scale; **POS**: Palliative Outcome Scale; **QoL**: quality of life; **RCT**: randomized controlled trial; **Self-PAC**: Self-Perceived Pain Assessment Knowledge and Confidence Scale; **SMD**: standardized mean difference.

## Data Availability

The original contributions presented in this study are included in the article/Supplementary Materials. Further inquiries can be directed to the corresponding author.

## Supplementary Materials

The following supporting information can be downloaded at https://www.mdpi.com/article/10.3390/curroncol33060312/s1, Table S1: Preferred Reporting Items for Systematic reviews and Meta-Analyses extension for Scoping Reviews (PRISMA-ScR) Checklist; Table S2: Search string strategy in databases.

## Abbreviations

The following abbreviations are used in this manuscript:

ACP	advanced care planning
AD	advanced directive
adj. OR	adjusted odds ratio
ASCO	American Society of Clinical Oncology
ASD	antiseizure drug
CCMB	Cancer Care Manitoba
CI	confidence interval
CNS	clinical nurse specialist
EANO	European Association of Neuro-Oncology
EHR	electronic health records
EOLC	end-of-life conversation
EORTCQLQ-C30	European organization for research and treatment of cancer quality of life questionnaire
EPC	early palliative care
EPCS	end-of-life professional caregiver survey
ESAS	Edmonton symptom assessment scale
ESMO	European Society of Medical Oncology
FACT-G	Functional Assessment of Cancer Therapy—General Measure
ICU	intensive care unit
OR	odds ratio
PC	palliative care
PCPS	Palliative Care Self-Reports Practices Scale
PHQ-9	Patient Health Questionnaire-9
POS	palliative outcome scale
QoL	quality of life
RCT	randomized controlled trial
Self-PAC	Self-Perceived Pain Assessment Knowledge and Confidence Scale
SMD	Standardized mean difference
