# Supplementary material for: Nursing Roles in Early Integration of Palliative and Supportive Care for Adults with Advanced Cancer: A Scoping Review
Source: Curr Oncol. 2026 May 27;33(6):312. doi: 10.3390/curroncol33060312 (PMC13298150; doi:10.3390/curroncol33060312)
Supplement: Supplementary file 1 [file curroncol-33-00312-s001.zip › curroncol-4229374-supplementary.pdf]

Table S1. Preferred Reporting Items for Systematic reviews and Meta-Analyses extension for Scoping Reviews (PRISMA-ScR) Checklist.

SECTION	ITEM	PRISMA-ScR CHECKLIST ITEM	REPORTED ON PAGE #
**TITLE**			
Title	1	Identify the report as a scoping review.	Title page
**ABSTRACT**			
Structured summary	2	Provide a structured summary that includes (as applicable): background, objectives, eligibility criteria, sources of evidence, charting methods, results, and conclusions that relate to the review questions and objectives.	Abstract
**INTRODUCTION**			
Rationale	3	Describe the rationale for the review in the context of what is already known. Explain why the review questions/objectives lend themselves to a scoping review approach.	Introduction, first and second paragraphs
Objectives	4	Provide an explicit statement of the questions and objectives being addressed with reference to their key elements (e.g., population or participants, concepts, and context) or other relevant key elements used to conceptualize the review questions and/or objectives.	Introduction, last paragraph (stating the aim and research question)
**METHODS**			
Protocol and registration	5	Indicate whether a review protocol exists; state if and where it can be accessed (e.g., a Web address); and if available, provide registration information, including the registration number.	Methods, Study design (2.1), last sentence (methods defined a priori; protocol not registered)
Eligibility criteria	6	Specify characteristics of the sources of evidence used as eligibility criteria (e.g., years considered, language, and publication status), and provide a rationale.	Methods, Conceptual focus and eligibility criteria (2.2, including Table 1)
Information sources*	7	Describe all information sources in the search (e.g., databases with dates of coverage and contact with authors to identify additional sources), as well as the date the most recent search was executed.	Methods, Study selection and search strategy (2.2, paragraph describing databases and date range) and Supplementary Table S2
Search	8	Present the full electronic search strategy for at least 1 database, including any limits used, such that it could be repeated.	Supplementary Table S2 (full electronic search strategies)
Selection of sources of evidence†	9	State the process for selecting sources of evidence (i.e., screening and eligibility) included in the scoping review.	Methods, Study selection (2.3) and Figure 1 (PRISMA-ScR flow diagram)
Data charting process‡	10	Describe the methods of charting data from the included sources of evidence	Methods, Data charting (2.4, description of standardized

SECTION	ITEM	PRISMA-ScR CHECKLIST ITEM	REPORTED ON PAGE #
		(e.g., calibrated forms or forms that have been tested by the team before their use, and whether data charting was done independently or in duplicate) and any processes for obtaining and confirming data from investigators.	form, piloting, and verification)
Data items	11	List and define all variables for which data were sought and any assumptions and simplifications made.	Methods, Data charting (2.4, paragraph listing extracted variables)
Critical appraisal of individual sources of evidence§	12	If done, provide a rationale for conducting a critical appraisal of included sources of evidence; describe the methods used and how this information was used in any data synthesis (if appropriate).	Methods, Synthesis of results (2.5, paragraph explaining that no formal risk-of-bias assessment was conducted)
Synthesis of results	13	Describe the methods of handling and summarizing the data that were charted.	Methods, Synthesis of results (2.5, description of thematic grouping into six analytical domains)
**RESULTS**			
Selection of sources of evidence	14	Give numbers of sources of evidence screened, assessed for eligibility, and included in the review, with reasons for exclusions at each stage, ideally using a flow diagram.	Results, Study selection and characteristics (3.1), first paragraph, and Figure 1
Characteristics of sources of evidence	15	For each source of evidence, present characteristics for which data were charted and provide the citations.	Results, Study selection and characteristics (3.1) and Table 2
Critical appraisal within sources of evidence	16	If done, present data on critical appraisal of included sources of evidence (see item 12).	Results, brief note in 3.1 that formal quality appraisal was not performed (linked to Methods 2.5)
Results of individual sources of evidence	17	For each included source of evidence, present the relevant data that were charted that relate to the review questions and objectives.	Results, subsections 3.2–3.6 and Table 2 (individual study findings)
Synthesis of results	18	Summarize and/or present the charting results as they relate to the review questions and objectives.	Results, second paragraph of 3.1 (six analytical domains) and Table 3, plus Figure 2
**DISCUSSION**			
Summary of evidence	19	Summarize the main results (including an overview of concepts, themes, and types of evidence available), link to the review questions and objectives, and consider the relevance to key groups.	Discussion, opening paragraphs summarizing six domains and main findings
Limitations	20	Discuss the limitations of the scoping review process.	Discussion, paragraph explicitly describing methodological and evidence

SECTION	ITEM	PRISMA-ScR CHECKLIST ITEM	REPORTED ON PAGE #
			limitations of the scoping review
Conclusions	21	Provide a general interpretation of the results with respect to the review questions and objectives, as well as potential implications and/or next steps.	Conclusions section (final paragraph of the manuscript)
**FUNDING**			
Funding	22	Describe sources of funding for the included sources of evidence, as well as sources of funding for the scoping review. Describe the role of the funders of the scoping review.	End of manuscript (Funding/Declarations section)

Table S2: Search string strategy in databases.

Database	Search String	Filters / Limits	Date of Search	Results
PubMed	(((“Neoplasms”[MeSH Terms]) OR (“Cancer”[Title/Abstract]) OR (“Oncology”[Title/Abstract]) OR (“Advanced cancer”[Title/Abstract]) OR (“Metastatic cancer”[Title/Abstract]) OR (“Malignancy”[Title/Abstract])) AND ((“Palliative Care”[MeSH Terms]) OR (“Supportive Care”[Title/Abstract]) OR (“Early palliative care”[Title/Abstract]) OR (“Terminal Care”[MeSH Terms]) OR (“Hospice Care”[MeSH Terms]) OR (“Palliative Medicine”[Title/Abstract]) OR (“End of life care”[Title/Abstract])) AND ((“Oncology Nursing”[MeSH Terms]) OR (“Hospice and Palliative Care Nursing”[MeSH Terms]) OR (“Nurse-led”[Title/Abstract]) OR (“Nursing role”[Title/Abstract]) OR (“Nursing care”[Title/Abstract]) OR (“Nursing intervention”[Title/Abstract]) OR (“Advance care planning”[Title/Abstract]) OR (“Nurse navigator”[Title/Abstract]) OR (“Clinical nurse specialist”[Title/Abstract]) OR (“Symptom management”[Title/Abstract])))	English language; Publication date: 2016/01/01–2025/11/30; Humans; Adults (≥18 years)	November 2025	318
CINAHL	((MH “Neoplasms+”) OR (TI “cancer” OR AB “cancer”) OR (TI “oncology” OR AB “oncology”) OR (TI “advanced cancer” OR AB “advanced cancer”) OR (TI “metastatic cancer” OR AB “metastatic cancer”) OR (TI “malignancy” OR AB “malignancy”)) AND ((MH “Palliative Care”) OR (MH “Terminal Care+”) OR (MH “Hospice Care”) OR (TI “palliative care” OR AB “palliative care”) OR (TI “supportive care” OR AB “supportive care”) OR (TI “early palliative care” OR AB “early palliative care”) OR (TI “end of life care” OR AB “end of life care”)) AND ((MH “Oncology Nursing”) OR (MH “Hospice and Palliative Nursing”) OR (TI “nurse-led” OR AB “nurse-led”) OR (TI “nursing role” OR AB “nursing role”) OR (TI “nursing care” OR AB “nursing care”) OR (TI “nursing intervention” OR AB “nursing intervention”) OR (TI “advance care planning” OR AB “advance care planning”) OR (TI “nurse navigator” OR AB “nurse navigator”) OR (TI “symptom management” OR AB “symptom management”))	English language; Published January 2016–November 2025; Peer reviewed; Adults	November 2025	184

Scopus	TITLE-ABS-KEY((“cancer” OR “oncology” OR “neoplasm*” OR “advanced cancer” OR “metastatic cancer” OR “malignancy”) AND (“palliative care” OR “supportive care” OR “early palliative care” OR “terminal care” OR “hospice care” OR “palliative medicine” OR “end of life care”) AND (“nurse-led” OR “nursing role” OR “nursing care” OR “nursing intervention” OR “oncology nursing” OR “advance care planning” OR “nurse navigator” OR “clinical nurse specialist” OR “symptom management” OR “palliative care nursing”))	English language; Date range 2016–2025; Document type: Article, Review; Subject area: Nursing, Medicine	November 2025	267
ScienceDirect	(“cancer” OR “oncology” OR “advanced cancer” OR “metastatic cancer” OR “neoplasm” OR “malignancy”) AND (“palliative care” OR “supportive care” OR “early palliative care” OR “terminal care” OR “hospice care” OR “end of life care”) AND (“nurse-led” OR “nursing role” OR “nursing care” OR “nursing intervention” OR “advance care planning” OR “oncology nursing” OR “nurse navigator” OR “symptom management”)	English language; Year range 2016–2025; Article type: Research articles, Review articles; Subject: Nursing and Health Professions, Medicine and Dentistry	November 2025	151
